# A systematic review of experimental methods to manipulate secondary hyperalgesia in humans: protocol

**DOI:** 10.1186/s13643-019-1120-7

**Published:** 2019-08-19

**Authors:** Victoria J. Madden, Gillian J. Bedwell, Prince C. Chikezie, Andrew S. C. Rice, Peter R. Kamerman

**Affiliations:** 1 Pain Unit, Department of Anaesthesia and Perioperative Medicine, Neuroscience Institute, University of Cape Town, D23.30 Groote Schuur Hospital, Observatory, Cape Town, 7925 South Africa; 2 Department of Psychiatry and Mental Health, University of Cape Town, Cape Town, South Africa; 3 Department of Health and Rehabilitation Sciences, University of Cape Town, Cape Town, South Africa; 4 School of Physiology, Faculty of Health Sciences, University of the Witwatersrand, Johannesburg, South Africa; 5 Pain Research Group, Department of Surgery and Cancer, Faculty of Medicine, Imperial College London, London, UK; 6 School of Pharmacy and Biomedical Sciences, Faculty of Health Sciences, Curtin University, Perth, Australia

**Keywords:** Hyperalgesia, Systematic review, Healthy volunteers, Quantitative sensory testing, Behaviour control

## Abstract

**Background:**

Neuropathic pain affects 7–10% of people, but responds poorly to pharmacotherapy, indicating a need for better treatments. Mechanistic research on neuropathic pain frequently uses human surrogate models of the secondary hyperalgesia that is a common feature of neuropathic pain. Experimentally induced secondary hyperalgesia has been manipulated with pharmacological and non-pharmacological methods to clarify the relative contributions of different mechanisms to secondary hyperalgesia. However, this literature has not been systematically synthesised. The aim of this systematic review is to identify, describe, and compare methods that have been used to manipulate experimentally induced secondary hyperalgesia in healthy humans.

**Methods:**

A systematic search strategy will be supplemented by reference list checks and direct contact with identified laboratories to maximise the identification of data reporting the experimental manipulation of experimentally induced secondary hyperalgesia in healthy humans. Duplicated screening, risk of bias assessment, and data extraction procedures will be used. Authors will be asked to provide data as necessary. Data will be pooled and meta-analyses conducted where possible, with subgrouping according to manipulation method. Manipulation methods will be ranked for potency and risk.

**Discussion:**

The results of this review will provide a useful reference for researchers interested in using experimental methods to manipulate secondary hyperalgesia in humans and will help to clarify the relative contributions of different mechanisms to secondary hyperalgesia.

**Systematic review registration:**

This protocol will be registered on PROSPERO before the review begins. Review records will be updated on PROSPERO once the review is complete. This review is intended for publication in a peer-reviewed journal. Analyses and scripts will be made publicly available.

**Electronic supplementary material:**

The online version of this article (10.1186/s13643-019-1120-7) contains supplementary material, which is available to authorized users.

## Introduction

### Rationale

Neuropathic pain (NP) currently affects 6.9–10% of the general population [1] and has severe consequences for the individual and for society. In a multicentre cohort survey, 2 in 3 people with NP had suboptimal sleep, 1 in 3 had a current mood disorder, over 90% reported feeling “sadness most of the time” and “tired most of the time”, and 18% had a current risk of suicide [2]. These findings are consistent with other reports that NP is associated with decreased life participation, low health-related quality of life, poor mental health, poor physical function, and higher use of health care services [3, 4]. The problem is only perpetuated by the fact that NP treatments perform poorly: the numbers needed to treat for even the first-line drugs range from 3.6 to 7.7 [5]. Given the severe consequences of NP and the obvious need for better treatments, understanding the mechanisms of NP is an important priority for health care research.

Secondary hyperalgesia—increased pain to a stimulus that is normally painful, in the area outside that of tissue damage or stimulation—is a common feature of NP [6]. Although a single model cannot account for all the possible features of clinical NP, experimentally induced secondary hyperalgesia has been widely used as a laboratory-based human surrogate model in studies that aim to elucidate the mechanisms that underlie neuropathic pain [7–11]. Methods that have been used to induce this secondary hyperalgesia in the experimental context include the application of capsaicin (intradermal or topical), topical mustard oil, topical menthol, repetitive heating, and high-frequency electrical stimulation [6, 9]. While secondary hyperalgesia is typically considered the primary outcome of studies that use these inductions, secondary outcomes may include primary hyperalgesia, secondary allodynia, and the surface area of secondary hyperalgesia. These outcomes can be compared to identify the relative contributions of different mechanisms. For example, while capsaicin-induced primary hyperalgesia could be attributed to changes in both peripheral and central nervous system processing, the broader distribution of secondary hyperalgesia suggests that it is mediated by changes in central processing [12].

Individual responses to an induction of secondary hyperalgesia can be useful for identifying certain profiles of sensitivity and factors that may be linked to those profiles. For example, in a small pilot study, women with a history of trauma showed a greater surface area of secondary hyperalgesia than women who did not have a history of trauma, after both groups underwent the same induction procedure [13]. This profiling may also have direct clinical application: the magnitude of induced secondary hyperalgesia could be useful for grouping patients by phenotype in order to better predict their responses to treatment [14].

As a surrogate model for NP, experimentally induced secondary hyperalgesia can be manipulated using pharmacological, psychological, or physical techniques, with a view to identifying potential treatments. The NMDA antagonist, Ketamine, has been found to reduce secondary hyperalgesia induced by capsaicin [15]. Negative expectations have been linked to greater secondary hyperalgesia induced by electrical stimulation [16], and an emotional disclosure intervention reduced the surface area of secondary hyperalgesia induced by capsaicin in women with a prior history of trauma [17]. A potential treatment that shows promise in animal studies can be tested in humans using the secondary hyperalgesia model as a step in the process towards refining and testing the treatment for clinical use.

Manipulating experimentally induced secondary hyperalgesia with interventions that have clinical potential is an important line of inquiry towards developing viable treatments for the relief of NP. However, although numerous studies have manipulated secondary hyperalgesia, there has been no attempt to systematically synthesise and appraise the literature on this topic.

### Aims and objectives

#### Aim

With this study, we aim to systematically identify, collate, and describe all the published studies that have applied manipulations intended to influence experimentally induced secondary hyperalgesia in healthy human participants. In doing so, we hope to provide a resource that will summarise the literature to date, provide pooled effect size estimates where possible, and identify gaps in knowledge and opportunities for further inquiry. Therefore, the primary aim of this systematic review is to identify, describe, and compare methods that have been used to manipulate experimentally induced secondary hyperalgesia in healthy humans.

#### Objectives

1. To identify methods used to manipulate secondary hyperalgesia
2. Describe each method in terms of procedure, essential equipment required, pain reported by participants during manipulation and harm reported in studies using each method
3. Determine the effect of each manipulation on
  - The magnitude of static mechanical secondary hyperalgesia
  - The surface area affected by secondary hyperalgesia

## Methods

### Eligibility criteria

#### Types of studies

We will include published and in-press or accepted records for which title, abstract, and full-text versions are available in English. Only prospective experimental studies will be included, that is, studies that attempted to manipulate secondary hyperalgesia for the purpose of studying the effects of the manipulation, and that did so in the context of an experiment, such that the secondary hyperalgesia was not a naturally occurring clinical phenomenon. In other words, participants must have begun the study without the secondary hyperalgesia being studied. To be eligible, studies must have assessed secondary hyperalgesia within 120 min after induction (so as not to miss the anticipated peak of the effect).

#### Types of study participants

We will include data from healthy human participants only. We place no restriction on the age of participants, but data from adults will be treated separately from data from children (< 18 years old).

#### Types of interventions

We will include data from experimental studies that aimed to manipulate secondary hyperalgesia (defined as “increased pain from a stimulus that normally provokes pain” [18] in an area adjacent to the stimulated area). Studies that manipulate secondary hyperalgesia as one step in a larger study will be considered eligible, provided that suitable baseline/control data are available to allow for estimation of the effect of the manipulation on secondary hyperalgesia.

#### Types of outcome measures

Pain will be defined as “an unpleasant sensory and emotional experience associated with actual or potential tissue damage, or described in terms of such damage” [18]. Pain must be measured by subjective self-report. Therefore, studies must have assessed the subjective (participant-reported) intensity of pain or sensation to somatosensory stimulation.

#### Primary outcome

The primary outcome is secondary hyperalgesia. Studies must have assessed mechanical secondary hyperalgesia (specifically, participant self-report to punctate mechanical stimulation) applied to the area surrounding the induction/manipulation site. Further, in order to qualify as “hyperalgesia”, the post-manipulation assessment must be compared to a within-subject control site (e.g. opposite limb on which induction but not manipulation was performed) or time point (e.g. after induction but before manipulation or, in the case of repeated inductions, to the same induction procedure performed without manipulation) or a between-subject control (e.g. group that underwent induction without the manipulation).

#### Secondary outcomes

- Surface area of secondary hyperalgesia, as measured using reproducible methods (such as a radial lines approach [11, 13, 15])
- Time course of secondary hyperalgesia
- Pain (except to test stimulation) reported during and after manipulation procedure
- Risks of the manipulation, defined as adverse events (e.g. skin damage, other adverse reaction)

### Search methods for identification of studies

#### Electronic searches

We will search the following electronic databases with a strategy that spans the time from their inception to the date of the search:

- Biosis (via Web of Science)
- PubMed (includes MEDLINE)
- Scopus
- ScienceDirect
- PsychArticles
- PsychInfo
- Cochrane library
- Web of Science Core (use to search and then use menu on left to filter for Core option and Biosis)

The search strategy will be:

((“human*” OR “women” or “woman” OR “man” OR “men” OR “participant*” OR “volunteer* OR individual*”)

OR

“normal skin” OR “healthy skin”)

AND

(“secondary hyperalgesia” OR “punctate hyperalgesia” OR “pinprick pain” OR “pinprick hyperalgesia” OR “mechanical hyperalgesia” OR “mechanical pain” OR “heat hyperalgesia” OR “neurogenic hyperalgesia”))

with all terms searched for in the title, keywords, or abstract.

+ limit to humans when possible in each database.

#### Other sources

We will check reference lists of eligible studies to check for other eligible studies not identified by electronic searching. We will also contact experts in the field and the corresponding authors of the most recent narrative reviews on experimental induction and manipulation of secondary hyperalgesia to ask for their assistance in identifying any missed studies (including Walter Magerl, Rolf Baron, Jürgen Sandkühler, Mark Wallace, Peter Drummond). We will also request any unpublished data from labs that have published extensively on these techniques, including data obtained during model development or optimisation.

### Data collection and analysis

#### Data management

The Systematic Review Facility (http://syrf.org.uk/) will be used to manage the review process.

#### Study selection

Two of three reviewers (VJM, GJB, and PC) will independently screen each identified record for eligibility in two sequential stages, screening (stage 1) title and abstracts and (stage 2) full texts. We will contact study authors a maximum of three times to obtain additional information that could influence eligibility. A customised eligibility form (see table below) will be used to record decisions in stage 2. Any disagreements about study inclusion will be resolved by discussion or by adjudication from a fourth, independent reviewer (PRK) if necessary. The study selection process will be reported using a PRISMA flow diagram.

Inclusion/exclusion criteria and grouping table

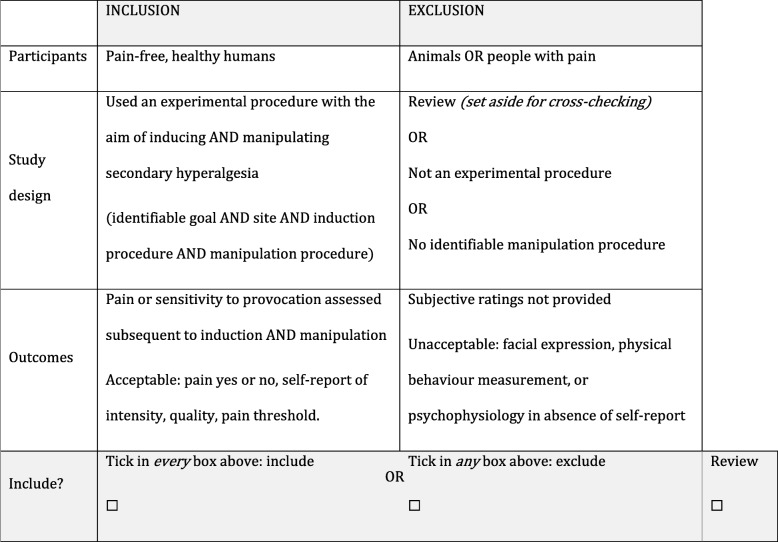



#### Assessment of risk of bias

A risk of bias assessment will be completed on each study. Two reviewers will independently appraise the risk of bias for the domains of selection, performance, detection, attrition, measurement, reporting, and other sources of bias. The criteria used to rate the risk of bias will be based on recommendations from the Cochrane collaboration [19] known quality instruments (e.g. the CONSORT [20] and STROBE [21] statements as relevant) and on known areas of bias relevant to the study design used [22], and are specified in the risk of bias assessment tool and guide (see Additional file 1). We will pilot the form on 2–3 studies and adapt it prior to formal application to all included studies. The focus of the risk of bias assessment will be on the risk that the data to be extracted to answer the questions of this review could be biased. The appraisals of the two independent reviewers will be compared and any disagreements resolved through discussion and consensus, or by third party adjudication if necessary.

#### Data extraction and management

Two of reviewers will independently extract data from each included study, using a standardised data extraction form. We will pilot and refine the data extraction form using 2–3 studies beforehand. We will contact study authors a maximum of 3 times to obtain required data that are unavailable or unclear from the published texts. If no reply is received within 6 weeks, we will consider the data unavailable. If the relevant data are not provided within 6 weeks of the first reply, we will consider the data unavailable for this review. Published data that seem implausible will be verified directly with the corresponding author where possible. Reviewers will resolve disagreements by discussion and consensus, or by a third party adjudication if necessary.

##### Study design

We will extract data on the study design, including the location/setting, date, sample size, primary aim, and outcomes measured.

##### Participants

We will extract data on the participants, including number, age, sex, pain status and sites of pain, co-morbid diagnoses, and psychological variables.

##### Interventions

We will extract data on the intervention(s) used to manipulate secondary hyperalgesia, including timing, duration, dosage (e.g. intensity or concentration), method of administration, modality, site, adverse effects, and ease of application where relevant. We will extract data on the equipment used (e.g. electrodes, needles, images, scripts). We will extract data on the control (site and/or time point and/or group). We will extract data on the manipulation check, where relevant (e.g. for psychological manipulations).

##### Outcomes

We will extract data on the test stimulus modalities, the scale or measure used to assess pain or change in perceived intensity, and the timing of assessments, that is, time from induction to first measurements and to each subsequent measurement, in minutes. We are interested in the following outcomes:

1. Secondary hyperalgesia intensity to calibrated punctate mechanical stimulation (e.g. Von Frey filament or calibrated pinprick stimulator)
2. The surface area affected by secondary hyperalgesia as tested in [1]
3. The duration of secondary hyperalgesia as tested in [1]

Where studies have included other outcomes associated with changes in secondary hyperalgesia (e.g. changes in brain activity shown on imaging), and when the secondary hyperalgesia data for that study indicate that the manipulation successfully influenced secondary hyperalgesia, we will discuss the findings as they pertain to the other outcomes.

##### Results

We will extract data on the participants in each study (including age, sex) the baseline and follow-up ratings for experimental and control sites for each participant or group, for each outcome. We will extract the number of participants who reported adverse effects and the number and natures of adverse effects reported.

#### Measures of intervention effects

When determining the potency of each manipulation method, our goal is to estimate the size of the effect of the manipulation by comparing the post-manipulation outcomes with the pre-manipulation outcomes and/or a control site/condition/group. For manipulations that are thought to have localised effects, a within-subject control site will be considered acceptable. For manipulations that are thought to have time-limited effects, a within-participant control time point will be acceptable. For manipulations that are thought to have systemic effects, a between-subject control or a control time-point (with suitable washout period) will be required. Data will be requested from authors where necessary.

#### Pooling of data

Data from different studies will be quantitatively pooled where possible and sensible, with subgrouping according to modality of induction/manipulation (e.g. negative expectation manipulation will be grouped separately to ketamine manipulation) and outcome (e.g. secondary hyperalgesia to mechanical punctate stimulation will be grouped separately to secondary allodynia to brush stroke), and with consideration given to measures of statistical heterogeneity (e.g. *I*^2^ statistic) and sensitivity analyses that exclude studies with particularly high risk of bias and studies in which obvious sources of methodological heterogeneity have been identified. This is not an individual patient data meta-analysis. We plan to use the RevMan software [23] or the R software with the package metafor to pool data and plot pooled data. The most up-to-date version of the relevant software package will be used. The outcome measure will be a standardised mean difference. We will use a random effects model to allow for anticipated heterogeneity between studies.

Where data exist but are unavailable, we plan to discuss the information that is available (e.g. number of participants tested, reasons for data unavailability, methods used) in narrative form, so as to mitigate against publication bias and provide a comprehensive summary of the literature available to answer the review question.

#### Relative ranking of interventions

If the quantity and quality of data allow, we will compare the pooled effect sizes (where available) to rank the different manipulations in the order of potency and risk. If the results reveal different potencies for the different outcomes of interest, then these ranked lists will be compiled separately for the different outcomes.

#### Publication bias

We will use funnel plots to examine for publication bias.

#### Assessment of the quality of the body of evidence

We will use the GRADE criteria [24] to assess the quality of the body of evidence for each manipulation, wherever more than one study is available for a certain manipulation.

## Publication and dissemination plan

We will update the review records on PROSPERO once the review is complete. We aim to publish this review in a peer-reviewed journal and to make all analyses and scripts publicly available.

## Reporting

Please see Additional file 2 PRISMA-P checklist for the elements included in this protocol.

## Additional file

Additional file 1: Risk of bias assessment tool. (DOCX 26 kb)

Additional file 2: PRISMA-P form. (DOC 85 kb)

## Data Availability

The public release of data used in this systematic review will depend on the ethics permissions to which the original data are subject. It is our preference to make data publicly available, but this may not be feasible due to existing restraints on the data. However, analysis scripts will be made publicly available for verification of the analytical processes.
