# Supplementary material for: A systematic review of experimental methods to manipulate secondary hyperalgesia in humans: protocol
Source: Syst Rev. 2019 Aug 19;8:208. doi: 10.1186/s13643-019-1120-7 (PMC6700765; doi:10.1186/s13643-019-1120-7)
Supplement: Supplementary file 1 — Risk of bias assessment tool. (DOCX 26 kb) [file 13643_2019_1120_MOESM1_ESM.docx]

| Article ID: Reviewer: | | |
| --- | --- | --- |
| **Selection bias** | | |
|  | Decision | Justification |
| Was the sampling/recruitment strategy appropriate to minimise bias? | € Yes € No € Unclear |  |
| Was it clearly and appropriately determined that participants were pain-free? | € Yes € No € Unclear |  |
| [B-G only] Similar baseline demographics among participants (age/sex/medical/psychological state)? | € Yes € No € Unclear |  |
| [Psych manip] Neutral psych status? | € Yes € No € Unclear |  |
| [B-G only] Random allocation  [B-site] Random allocation | € Yes € No € Unclear |  |
| **Risk of selection bias summary** | € High (failure to include any of the above probably influenced results FOR THE QUESTION OF THIS REVIEW)  € Low (results unlikely to have been influenced)  € Unclear (not enough information) | |

**Risk of bias assessment tool**

| **Performance bias** | | |
| --- | --- | --- |
| Blinding | Decision | Justification |
| Were participants blinded to the research question and paradigm and [if relevant] group allocation? | € Yes € No € Unclear |  |
| **Risk of performance bias summary** | € High    € Low  € Unclear | |
| **Detection bias** | | |
| Were outcome assessors blinded to the research question and paradigm? | € Yes € No € Unclear |  |
| Were analysing researchers blinded to the group allocation of participants and/or to site allocation? | € Yes € No € Unclear |  |
| **Risk of detection bias summary** | € High    € Low  € Unclear | |

| **Manipulation veracity** | | | | |
| --- | --- | --- | --- | --- |
| [Psych] Did a manipulation check confirm the effectiveness of the manipulation? | | € Yes € No € Unclear |  | |
| **Risk of manipulation veracity problem** | | € High € Low € Unclear |  | |
| **Attrition bias** | | | | |
| Incomplete outcome data | | Decision | Justification | |
| Have attrition/exclusions/ withdrawals been reported and appropriately dealt with in analysis? | | € Yes € No € Unclear |  | |
| **Risk of attrition bias summary** | | € High    € Low  € Unclear | | |
| **Measurement bias** | | | | |
|  | | Decision | Justification | |
| Were valid and reliable outcome measurements used to assess severity & SA of secondary hyperalgesia? | | 2H: € Yes € No € Unclear  SA: € Yes € No € Unclear |  | |
| Were identical equipment items used for measurements between groups/sites/time points? | | 2H: € Yes € No € Unclear  SA: € Yes € No € Unclear |  | |
| Did the same assessor conduct assessments between groups/sites/time points? | | 2H: € Yes € No € Unclear  SA: € Yes € No € Unclear |  | |
| **Risk of measurement bias summary** | | 2H: € Yes € No € Unclear  SA: € Yes € No € Unclear | | |
| **Reporting bias** | | | | |
| Selective reporting | | Decision | Justification | |
| Were all outcomes for experimental and control groups reported on? | | € Yes € No |  | |
| Were conflicts of interest and funding sources declared? | | € Yes € No |  | |
| **Risk of reporting bias summary** | | € High    € Low  € Unclear | | |
| **Risk of bias summary** | | | | |
| Risk of bias | Description | | | Study bias outcome |
| High risk of bias | Plausible bias that seriously weakens confidence in the results. | | |  |
| Low risk of bias | Plausible bias unlikely to seriously alter or diminish trust in the results. | | |  |
| Unclear risk of bias | Insufficient information available to make a judgement. | | |  |

Comments:

| Article ID: Reviewer: | | |
| --- | --- | --- |
| **Selection bias** | | |
|  | Decision | Justification |
| Was the sampling/recruitment strategy appropriate to minimise bias? | € Yes  € No  € Unclear | Yes: general population or subgroup. Convenience sampling is acceptable as long as eligibility criteria do not restrict to a certain group that could plausibly respond differently to the induction.  No: group selected on basis of particular feature (e.g. high catastrophising positive affect / athletes in training) |
| Was it clearly and appropriately determined that participants were pain-free? | € Yes  € No  € Unclear | Yes: participant self-report of no pain at time of testing AND no history of chronic pain (pain on most days for > 3 mo) in preceding 2 years.  No: reports failure to ask BOTH questions.  Unclear: does not report asking both questions. |
| [B-G only] Similar baseline demographics among participants (age/sex/medical/psychological state)? | € Yes  € No  € Unclear | Yes: Psych (trauma Hx, stress status, general affect, sex, age, medication variables accounted for and similar)  No: Psychiatric diagnoses or medication use (esp analgesics/anti-inflammatories/SNRI, etc) amongst participants.  Unclear: not reported  *Consider design features, e.g. within-subject control or pre-post design |
| [Psych manip] Neutral psych status? | € Yes  € No € Unclear | Yes: Psych variables accounted for and normal  No: selected for responses on psych assessment |
| [B-G only] Random allocation  [B-site] Random allocation | € Yes  € No  € Unclear | Yes: random sequence generation / roll of die / other truly random procedure named  No: counterbalancing of group size (i.e. pseudo-randomisation)[but consider ROB in context] / sequential allocation  Unclear: not reported in enough detail to allow decision |
| **Risk of selection bias summary** | € High (failure to include any of the above probably influenced results FOR THE QUESTION OF THIS REVIEW)  € Low (results unlikely to have been influenced)  € Unclear (not enough information) | |

**Guide to decision-making for risk of bias assessment**

| **Performance bias** | | |
| --- | --- | --- |
| Blinding | Decision | Justification |
| Were participants blinded to the research question and paradigm and [if relevant] group allocation? | € Yes  € No  € Unclear | Yes: evidence provided - blinding strategy AND blinding check AND results reported AND analysis done accordingly  No: Blinding reported broken  Unclear: not enough information / failure to report) |
| **Risk of performance bias summary** | € High  € Low  € Unclear | High: Plausible doubt that participant blinding was applied and maintained throughout  Low: Confident that participant blinding was applied and maintained throughout  Unclear: not enough information to make informed judgement (e.g. blinding strategy AND blinding check AND results mentioned BUT not fully reported) |
| **Detection bias** | | |
| Were outcome assessors blinded to the research question and paradigm? | € Yes  € No  € Unclear | Yes: evidence provided - blinding strategy AND blinding check AND results reported AND analysis done accordingly  No: Blinding reported broken  Unclear: not enough information / failure to report) |
| Were analysing researchers blinded to the group allocation of participants and/or to site allocation? | € Yes  € No  € Unclear | Yes: evidence provided - blinding strategy AND blinding check AND results reported AND analysis done accordingly  No: Blinding reported broken  Unclear: not enough information / failure to report) |
| **Risk of detection bias summary** | € High  € Low  € Unclear | High: Plausible doubt that participant blinding was applied and maintained throughout  Low: Confident that participant blinding was applied and maintained throughout  Unclear: not enough information to make informed judgement (e.g. blinding strategy AND blinding check AND results mentioned BUT not fully reported) |

| **Risk of manipulation veracity problem** | | | | | |
| --- | --- | --- | --- | --- | --- |
| [Psych] Did a manipulation check confirm the effectiveness of the manipulation? | | € Yes € No € Unclear | | Yes: manip check done and results reported and confirmed effectiveness  No: no manipulation check done OR manip check done but results not reported.  Unclear: manip check done and results confirmed ineffectiveness or were inconclusive | |
| **Risk of manipulation veracity problem** | | € High € Low € Unclear | |  | |
| **Attrition bias** | | | | | |
| Incomplete outcome data | | Decision | | Justification | |
| Have attrition/exclusions/ withdrawals been reported and appropriately dealt with in analysis? | | € Yes € No € Unclear | | Yes: no attrition/withdrawals OR stats handled withdrawals appropriately AND relevant adverse events reported | |
| **Risk of attrition bias summary** | | € High    € Low  € Unclear | | | |
| **Measurement bias** | | | | | |
|  | | Decision | | Justification | |
| Were valid and reliable outcome measurements used to assess severity & SA of secondary hyperalgesia? | | 2H: € Yes € No € Unclear  SA: € Yes € No € Unclear | | Yes:  Self-report: VAS / NRS / validated scale  Surface area: independently duplicated measurements or validated approach  Consider test-retest reliability if relevant  No: single measurement of distance/SA; un-validated self-report scale | |
| Were identical equipment items used for measurements between groups/sites/time points? | | 2H: € Yes € No € Unclear  SA: € Yes € No € Unclear | |  | |
| Did the same assessor conduct assessments between groups/sites/time points? | | 2H: € Yes € No € Unclear  SA: € Yes € No € Unclear | |  | |
| **Risk of measurement bias summary** | | 2H: € Yes € No € Unclear  SA: € Yes € No € Unclear | | | |
| **Reporting bias** | | | | | |
| Selective reporting | | | Decision | Justification | |
| Were all outcomes for experimental and control groups reported on? | | | € Yes € No | Check each outcome (compare methods vs results) | |
| Were conflicts of interest and funding sources declared? | | | € Yes € No | Consider relevant conflicts | |
| **Risk of reporting bias summary** | | | € High    € Low  € Unclear | | |
| **Risk of bias summary** | | | | | |
| Risk of bias | Description | | | | Study bias outcome |
| High risk of bias | Plausible bias that seriously weakens confidence in the results. | | | |  |
| Low risk of bias | Plausible bias unlikely to seriously alter or diminish trust in the results. | | | |  |
| Unclear risk of bias | Insufficient information available to make a judgement. | | | |  |

Comments:
